# Neural mobilisation effects in nerve function and nerve structure of patients with peripheral neuropathic pain: A systematic review with meta-analysis

**DOI:** 10.1371/journal.pone.0313025

**Published:** 2024-11-08

**Authors:** Juliana Valentim Bittencourt, Leticia Amaral Corrêa, Maria Alice Mainenti Pagnez, Jéssica Pinto Martins do Rio, Gustavo Felicio Telles, Stephanie Mathieson, Leandro Alberto Calazans Nogueira

**Affiliations:** 1 Rehabilitation Science Postgraduate Program at Augusto Motta University Centre (UNISUAM), Rio de Janeiro, Brazil; 2 Department of Chiropractic, Faculty of Medicine, Health and Human Sciences, Macquarie University, Sydney, Australia; 3 Sydney Musculoskeletal Health, Faculty of Medicine and Health, Sydney School of Health Sciences, The Kolling Institute, The University of Sydney, Sydney, Australia; 4 Physiotherapy Department at Federal Institute of Rio de Janeiro (IFRJ), Rio de Janeiro, Brazil; University College Dublin - National University of Ireland: University College Dublin, IRELAND

## Abstract

**Objective:**

To assess the effects of neural mobilisation on nerve function and nerve structure of patients with peripheral neuropathic pain.

**Methods:**

A systematic review with meta-analysis was conducted. Medline, Embase, CINAHL, Cochrane Library, and World Health Organization International Clinical Trials Registry Platform were searched without restrictions. Eligibility criteria included controlled trials or quasi-experimental studies comparing neural mobilisation versus sham, active or inactive control in adults with peripheral neuropathic pain. Primary outcomes were the change in peripheral nerve cross-sectional area. Secondary outcomes included nerve echogenicity, nerve excursion and nerve conduction. Random effects meta-analysis was conducted. Risk of bias was assessed with the Cochrane Collaboration tool, and certainty of evidence was assessed using the Grading of Recommendations Assessment, Development, and Evaluation framework.

**Results:**

Eleven randomised controlled trials and four quasi-experimental studies (total sample = 722 participants) were included. Thirteen studies included participants with carpal tunnel syndrome. Two studies examined the cross-sectional area, revealing improvements (i.e., a reduction) in the cross-sectional area after the neural mobilisation. Neural mobilisation improved motor [mean difference = 2.95 (95%CI 1.67 to 4.22)] and sensory conduction velocity in short-term [mean difference = 11.74 (95%CI 7.06 to 16.43)], compared to control. Neural mobilisation did not alter distal motor or sensory latency.

**Conclusion:**

Neural mobilisation seems to improve (i.e., a reduced) the cross-sectional area (very low-quality evidence) and sensory conduction velocity (very low-quality evidence). Neural mobilisation was superior to control in improving motor conduction velocity in patients with peripheral neuropathic pain with moderate quality evidence. Distal motor or sensory latency presented similar results compared to other interventions. Our findings should be interpreted cautiously since most studies included patients with carpal tunnel syndrome.

## Introduction

Neuropathic pain is a significant cause of suffering and disability. The prevalence of chronic neuropathic pain ranges between 7% and 10% of the general population [1]. A neuropathic component is estimated to be in approximately one-third of the pain syndromes [2]. Neuropathic pain can be associated with musculoskeletal conditions, such as low back pain [3], whiplash disorders [4, 5], lateral epicondylalgia [6], and carpal tunnel syndrome [7]. Although neuropathic pain is commonly observed in musculoskeletal conditions, there is an inconsistent use of its terminology for diagnostic labels in clinical practice and scientific literature [8]. Peripheral neuropathic pain could be present as radicular pain without (e.g., pain travelling to the limbs in regions that are similar to dermatomes but not entirely identical) or with radiculopathy (e.g., pain alongside neurological deficits in dermatomal or myotomal distribution or impaired reflexes) [8]. Therefore, the complex nature of peripheral neuropathic pain underscores the need for tailored treatment approaches to address its multifaceted challenges.

Clinical guidelines and consensus statements recommend pharmacologic management as treatment for patients with neuropathic pain [9–12], including tricyclic antidepressants, serotonin-norepinephrine reuptake inhibitors, and gabapentin as first-line treatments [9, 10, 13]. Clinical guidelines also recommend non-pharmacological approaches, such as conservative treatments like exercise and manual therapy [14]. In cases where patients do not respond adequately to previous treatments, invasive procedures may be considered [15]. Given the adverse effects caused by pharmacological interventions, the recommendations available are still inconsistent [16]. Thus, effective and safe approaches are needed for patients with peripheral neuropathic pain.

Neural mobilisation is used to reach the neural structures or surrounding tissue and can be performed manually [17, 18]. Neural mobilisation promotes clinical benefits for patients with nerve-related conditions [19–21]. For instance, neural mobilisation benefits back and neck pain patients [19]. Similarly, neural mobilisation showed moderate effects on the joint flexibility of healthy participants and large effects on pain intensity and disability in low back pain [20]. Moreover, neural mobilisation showed moderate to large positive results on pain intensity and disability in musculoskeletal disorders patients [22]. Previous studies have also shown that neural mobilisation reduces intraneural oedema [23] and improves intraneural fluid dispersion [24, 25]. There was a simultaneous increase in the magnitude of neural adaptive movement with a straight leg elevation test and the resolution of the radicular and low back pain symptoms [26]. Although high-quality evidence demonstrates the clinical benefit of the neural mobilisation techniques, the effects of the method on nerve function and structure have not yet been adequately explored and summarised.

Peripheral nerves and their mechanical properties have been studied extensively. Healthy peripheral nerves present a tubular form, alternating hypoechogenic and hyperechogenic zones corresponding to nerve and perineural fibres visible on ultrasonography imaging (USI) [27]. Changes in nerve structure are commonly observed in patients with peripheral neuropathies. For instance, patients with carpal tunnel syndrome showed an increase in the cross-sectional area of the median nerve, increased nerve swelling at the wrist, nerve hypoechogenicity, disturbance of the fascicular structure, reduced nerve slipping, and increased vascularity [28]. Similarly, patients with fibular nerve entrapment neuropathy demonstrated an increase in the cross-sectional area of the nerve and an increased fibular to popliteal fossa swelling ratio [28]. Several instruments have been used to assess peripheral nerve structure and function. Nerve conduction tests (i.e., electroneuromyography (ENMG)) and imaging exams (i.e., USI and magnetic resonance imaging (MRI)) are most commonly used. The cross-sectional area and echogenicity of the peripheral nerves can be quantified by USI [28, 29]. ENMG may be used in the classification of neuropathies [30] in the assessment of nerve conduction [31], and ENMG findings are correlated with structural abnormalities in the nerve [32]. The USI usually measures the excursion of the peripheral nerves [33, 34]. Also, the MRI method has been used in peripheral neuropathies to offer more quantitative features [35], such as nerve volume, cross-sectional area, diffusion properties of water molecules along the nerve fibres, and the presence of oedema or inflammation. This systematic review aimed to assess the effects of neural mobilisation on nerve function and nerve structure of patients with peripheral neuropathic pain.

## Materials and methods

### Protocol and registration

A systematic review was reported following the Preferred Reporting Items for Systematic Reviews and Meta-Analyses (PRISMA) guidelines [36] (See S1 File). The protocol was registered in advance with the international Prospective Register of Systematic Reviews (PROSPERO registration number: CRD42022337067).

### Data sources and searches

We performed electronic searches of Medline, Embase, Cumulative Index to Nursing and Allied Health Literature, and the Cochrane Central Register of Controlled Trials. We performed the initial electronic search from inception to 1^st^ November 2023 without restrictions on language, publication period, or publication status. We used keywords, Medical Subject Headings (MeSH), and other index terms, as well as combinations of these terms and appropriate synonyms across all included databases. The Medline search strategy is provided in the S2 File.

We searched clinical trial databases (ClinicalTrials.gov, World Health Organization International Clinical Trials Registry Platform (apps.who.int/trialsearch/) to identify potentially eligible additional published or unpublished data. We conducted manual search of the reference lists of included studies and previous systematic reviews related to this topic for any potentially eligible studies.

### Eligibility criteria

We included controlled trials or quasi-experimental studies, which assessed the neurophysiological effects of neural mobilisation in patients with peripheral neuropathic pain, including radicular pain with or without radiculopathy. Participants were adults (aged 18 years or over) with one or more clinically diagnosed peripheral neuropathic pain (e.g., carpal tunnel syndrome, sciatica, cubital tunnel syndrome, low back pain with radicular symptoms, cervicobrachial pain). As diagnostic criteria for peripheral neuropathic pain varies in the literature, we considered studies that defined peripheral neuropathic pain via clinical diagnosis, nerve conduction studies, or imaging exams. We included studies that used slider or tensioner techniques as treatment. Neurodynamic tests (e.g., straight leg raises, slump test and upper limb neurodynamic tests) are examples of movements used in the sliders and tensioners techniques. We considered studies with neural mobilisation prescribed or performed by a health professional and with any duration of treatment or follow-up. The eligible comparison conditions included sham neural mobilisation or active (e.g., walking, aerobics exercises, stretching exercises, balance training, tai chi, yoga, Pilates) or inactive control (e.g., usual care, wait-list control, education booklets, education group, telephone counselling, storytelling).

Some conditions were excluded, such as those related to metabolic disorders (e.g., peripheral diabetic neuropathy), neuropathies associated with viral infections (e.g., post-herpetic neuralgia, HIV, leprosy) and chemotherapy-induced peripheral neuropathies. Moreover, studies were excluded if participants had non-specific or mechanical spinal pain, central spinal canal stenosis, cerebral palsy, paraplegia or quadriplegia, and other major conditions (e.g., fractures, dislocations). We did not include editorials, comments, letters, correspondence, abstracts, case reports, clinical observations, reviews, or studies with animals.

### Study selection

Records found through searching were exported to EndNote reference management software (version X9), and two independent review authors (J.V.B. and L.A.C.) screened all search results for potentially eligible studies (See S3 File). Potentially eligible articles based on the title, abstract, and full text were sequentially screened. A third independent review author (L.A.C.N) resolved any disagreement about eligibility (See S1 Table).

### Data extraction

We extracted data from each included study using a standardised extraction form proforma. Two independent review authors (J.V.B. and L.A.C.) extracted all data, and a third author (L.A.C.N.) revised the data in case of disagreements. The data extracted included details about the study characteristics (i.e., authors, publication year, and country of origin), study design, participant characteristics (i.e., number of participants and clinical condition), detailed treatment performed, control group information, outcomes, follow-up time points, primary results, and conclusions. We extracted pre-treatment and post-treatment means, standard deviations, and 95% confidence intervals for outcomes of interest. We obtained data from the trial registry where data were not available in the published manuscript. The authors were contacted in the event of missing data.

### Outcomes measures

The primary outcome measures were the nerve structure, such as a reduction in the cross-sectional area of the nerve measured by USI, MRI, or other imaging exams.

The secondary outcome measure of nerve structure was echogenicity, as measured by USI. We were also interested in the effects on nerve function, explicitly improving nerve excursion (measured by USI or other imaging exams) and nerve conduction (measured by electromyography or other nerve conduction tests). We categorised follow-up outcome data of individual studies into short-term outcomes (defined as those occurring in less than 3 months), intermediate outcomes (between 3 and 12 months), and long-term outcomes (as those occurring more than 12 months after randomisation).

### Data synthesis

We calculated changes from the baseline. We used Cochrane’s RevMan calculator to estimate the change from baseline standard deviations, where they were not reported.

Meta-analysis was conducted when an outcome was reported in two or more studies. In cases where meta-analysis was not possible, descriptive analyses were performed. The studies were grouped according to the similarity of the outcomes, and it was not necessary to convert the values to a common metric.

### Data analysis

The flow of studies was summarised in a study flow diagram following the PRISMA statement [36]. Study characteristics were reported descriptively. Continuous outcomes are presented as mean differences (MDs) with 95% confidence intervals (CIs) between the intervention and control groups. The meta-analysis was performed using a random effects model (See S2 Table). The heterogeneity analysis was performed using the I^2^ values and considered as moderate I^2^ value of 30% to 60%, substantial 50% and 90%, and considerable heterogeneity in values more than 75%, following The Cochrane Handbook of Systematic Reviews of Interventions recommendations [37].

### Risk of bias and certainty of evidence

We assessed the risk of bias using the original Cochrane Risk of Bias (ROB) tool for randomised trials [38] and the Risk of Bias in Non-randomised Studies (ROBINS-I) tool for studies that did not use randomisation to allocate interventions [39]. The classification of the ROB tool includes seven items assessing the risk of bias: selection bias (random sequence generation and allocation concealment), performance bias (blinding of participants), detection bias (blinding of outcome assessment), attrition bias (incomplete outcome data), reporting bias (selective reporting), and other sources of biases. The judgment for each item was classified as low risk, high risk or unclear risk of bias [38]. ROBINS-I tool includes seven items assessing the risk of bias in domains: bias due to confounding, bias in the selection of participants into the study, bias in classification of interventions, bias due to departures from intended interventions, bias due to missing data, bias in the measurement of outcomes, and bias in the selection of reported results. Despite the availability of an updated version of the Cochrane risk-of-bias tool for randomised trials (RoB 2), we choose to use the ROB, as RoB 2 presents challenges with low interrater reliability in its application [40]. Two reviewers (JVB and LAC) assessed the risk of bias for each study, and a third reviewer (LACN) revised it in case of disagreements.

The overall quality of evidence was assessed by the Grading of Recommendations Assessment Development and Evaluation (GRADE) [41]. We considered the following items: study design, risk of bias, imprecision, indirectness, inconsistency, and publication bias. The overall quality of evidence per outcome was determined as high, moderate, low, or very low. We present a summary of the overall strength of evidence available using the GRADE Summary of Findings table produced using GRADEproGTD (https://www.gradepro.org/).

## Results

**Fig 1:**
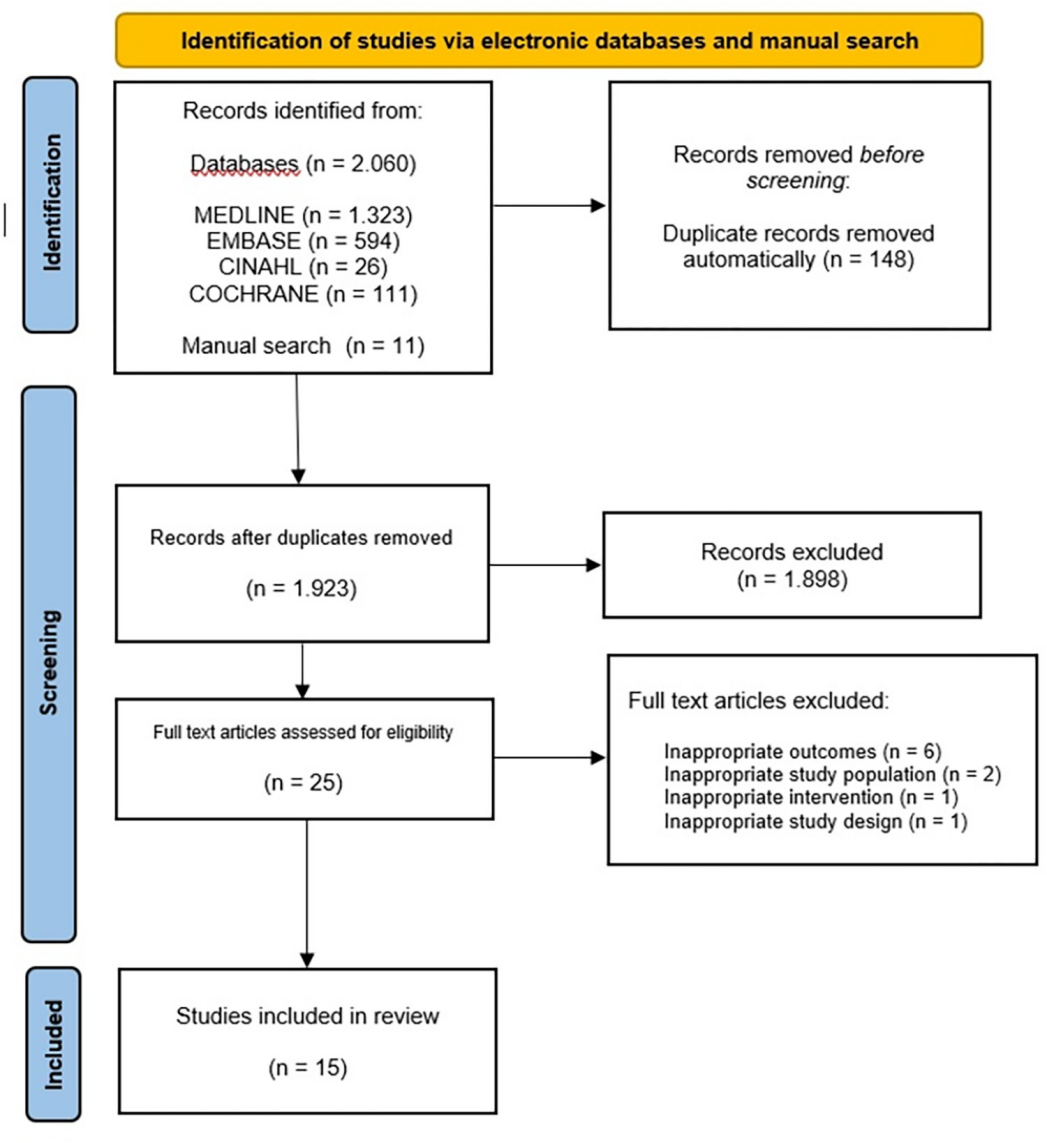
Flow diagram of search results and studies included.

The database search retrieved 2,060records, and the manual search retrieved ten. Of these, we selected 25 for full-text assessment. A total of 15 studies (11 controlled trials and four quasi-experimental studies) fulfilled the inclusion criteria (Fig 1).

### Characteristics of the studies

Included studies were conducted in 9 different countries, namely Italy [42], Portugal [43], Canada [44], Turkey [7, 45, 46], United States [47], Australia [23], Sweden [48], Poland [49–51], and Iran [52–54]. The studies included were published between 2005 and 2023. Of these, two studies were published in 2009 [47, 48], two in 2018 [46, 50], two in 2019 [43, 51] and two in 2020 [44, 52]. Ten studies [7, 42, 43, 45–48, 52–54] used tensioning mobilisation techniques, three investigations [49–51] used sliding and tensioning techniques, and two studies [23, 44] used sliding mobilisation techniques. The characteristics of the included studies are presented in Table 1.

**Table 1:** Descriptions of studies on participants with peripheral neuropathic pain.

Study	Design	Country	Study population	Techniques	Intervention and comparator	Outcomes measured
**Pinar 2005** [45]	RCT	Turkey	CTS (n = 26)	Tension	Splint plus patient training program *or* splint plus patient training program plus neural mobilisation	Before and 10-week after intervention • NCS (electrophysiologic test): distal motor latency
**Baysal et al. 2006** [7]	RCT	Turkey	CTS (n = 36)	Tension	Splinting plus neural mobilisation or splinting plus ultrasound or splinting plus neural mobilisation plus ultrasound	Before, after intervention, and 8-week after intervention • NCS: motor latency, sensory latency
**Bialosky et al. 2009** [47]	RCT	United States	CTS (n = 40)	Tension	Neural mobilisation or sham technique	Before and after intervention • NCS (electrodiagnostic test): distal motor latency
**Svernlöv et al. 2009** [48]	RCT	Sweden	Cubital Tunnel Syndrome n = 70 (n = 39 women and n = 31 men)	Tension	Splinting plus information or neural mobilisation plus information or information	Before and 6-month after intervention • NCS: sensory conduction velocity, motor conduction velocity, electromyography
**Schmid et al. 2012** [23]	RCT	Australia	CTS (n = 20)	Sliding	Splinting or neural mobilisation home program	Before, 10-minute after intervention, and 1-week after intervention • Nerve structure evaluation: signal intensity
**Oskouei et al. 2014** [54]	RCT	Iran	CTS n = 20 (n = 16 hands in each group)	Tension	Routine physiotherapy (splint, TENS, and therapeutic ultrasound) or routine physiotherapy plus neural mobilisation	Before and 4-week after intervention • NCS: motor distal latency and sensory distal latency
**Ginanneschi et al. 2015** [42]	QES	Italy	CTS n = 16 [n = 8 hands (men = 1; women = 7) and 8 healthy participants]	Tension	Neural mobilisation	Before and after intervention • NCS: sensory conduction velocity, sensory action potential amplitude, distal motor latencies
**Wolny et al. 2017** [49]	RCT	Poland	CTS (n = 140)	Sliding and tension	Neural mobilisation plus functional massage plus bone mobilisations techniques or laser plus ultrasound therapy	Before and after intervention • NCS: sensory conduction velocity, motor conduction velocity, motor latency, standardized latency
**Yildirim et al. 2018** [46]	RCT	Turkey	CTS (n = 21)	Tension	Kinesiotaping plus neural mobilisation or neural mobilisation	Before, 3-week after intervention, and 6-week after intervention • Nerve structure evaluation: CSA
**Wolny & Linek, 2018** [50]	RCT	Poland	CTS (n = 150)	Sliding and tension	Neural mobilisation or “sham” therapy	Before and after intervention • NCS: sensory conduction velocity, motor conduction velocity, motor latency
**Neto et al. 2019** [43]	QES	Portugal	Sciatica n = 16 (n = 8 chronic sciatica and n = 8 health participants)	Tension	Neural mobilisation	Before and after intervention • Nerve structure evaluation: nerve stiffness (SWV)
**Wolny & Linek, 2019** [51]	RCT	Poland	CTS (n = 103)	Sliding and tension	Neural mobilisation or control group	NCS: Before and 1-month after treatment • NCS: sensory conduction velocity, motor conduction velocity, motor latency
**Paquette et al. 2020** [44]	QES	Canada	CTS (n = 14)	Sliding	Neural mobilisation home program plus videoconference plus logbook	Before and 1-week after the completion of a 4-week intervention program • NCS (US): nerve biological integrity, nerve mechanical properties
**Talebi et al. 2020** [52]	RCT	Iran	CTS (n = 30)	Tension	Nerve mobilisation or mechanical interface mobilisation	Before and immediately after the end of the treatment period • NCS: motor distal latency, sensory distal latency
**Khademi et al. 2023** [53]	QES	Iran	CTS (n = 20)	Tension	Neural mobilisation	Before and immediately after one session of neural mobilisation • Nerve structure evaluation: nerve stiffness • Nerve structure evaluation: CSA

Abbreviations: CSA = Cross-Sectional Area; CTS = Carpal Tunnel Syndrome; NCS = Nerve Conduction Studies; QES = Quasi-Experimental Study

RCT = Randomised Clinical Trials; SWV: Shear Wave Velocity; TENS = Transcutaneous Electrical Nerve Stimulation.

### Characteristics of interventions

A controlled trial compared neural mobilisation versus no treatment in 103 patients with carpal tunnel syndrome [51]. One study compared the effect of neural mobilisation in patients with carpal tunnel syndrome [53]. Similarly, another study compared the effect of neural mobilisation in patients with carpal tunnel syndrome and healthy participants [42]. Another study performed neural mobilisation in patients with sciatica and controls [43]. Two studies compared the effect of neural mobilisation in a group of patients with carpal tunnel syndrome with no comparison group [44]. Five studies compared a group of neural mobilisations versus other interventions [23, 45, 46, 49, 54], and two studies compared different regimes of neural mobilisation [7, 52] for participants with carpal tunnel syndrome. Two studies [47, 50] investigated the effects of neural mobilisation compared to the sham technique in participants with carpal tunnel syndrome. One study compared the impact of adding neural mobilisation to information versus other approaches with no neural mobilisation to participants with cubital tunnel syndrome [48].

Eight studies [42, 43, 49–54] offered neural mobilisation individually and in person, performed by a physiotherapist. Five studies [7, 23, 44, 45, 48] provided a neural mobilisation program that could be carried out at home. The neural mobilisation session lasted from 3 to more than 20 minutes. The frequency of neural mobilisation treatment ranged from only one session to seven sessions per week. Treatment periods varied between one session and 12 weeks.

### Outcomes

#### Cross-sectional area

Two studies examined the cross-sectional area [46, 53]. One study found improvements (i.e., a reduction) in the median cross-sectional area after neural mobilisation with or without kinesiotaping in patients with carpal tunnel syndrome. Both groups reduced the cross-sectional area in the short-term, but there was no statistically significant difference in the cross-sectional area between the groups [46]. One study reported a significant cross-sectional decrease in the median nerve immediately after the treatment of neural mobilisation in a non-randomised study [53].

#### Nerve motor conduction–Distal motor latency

**Fig 2:**
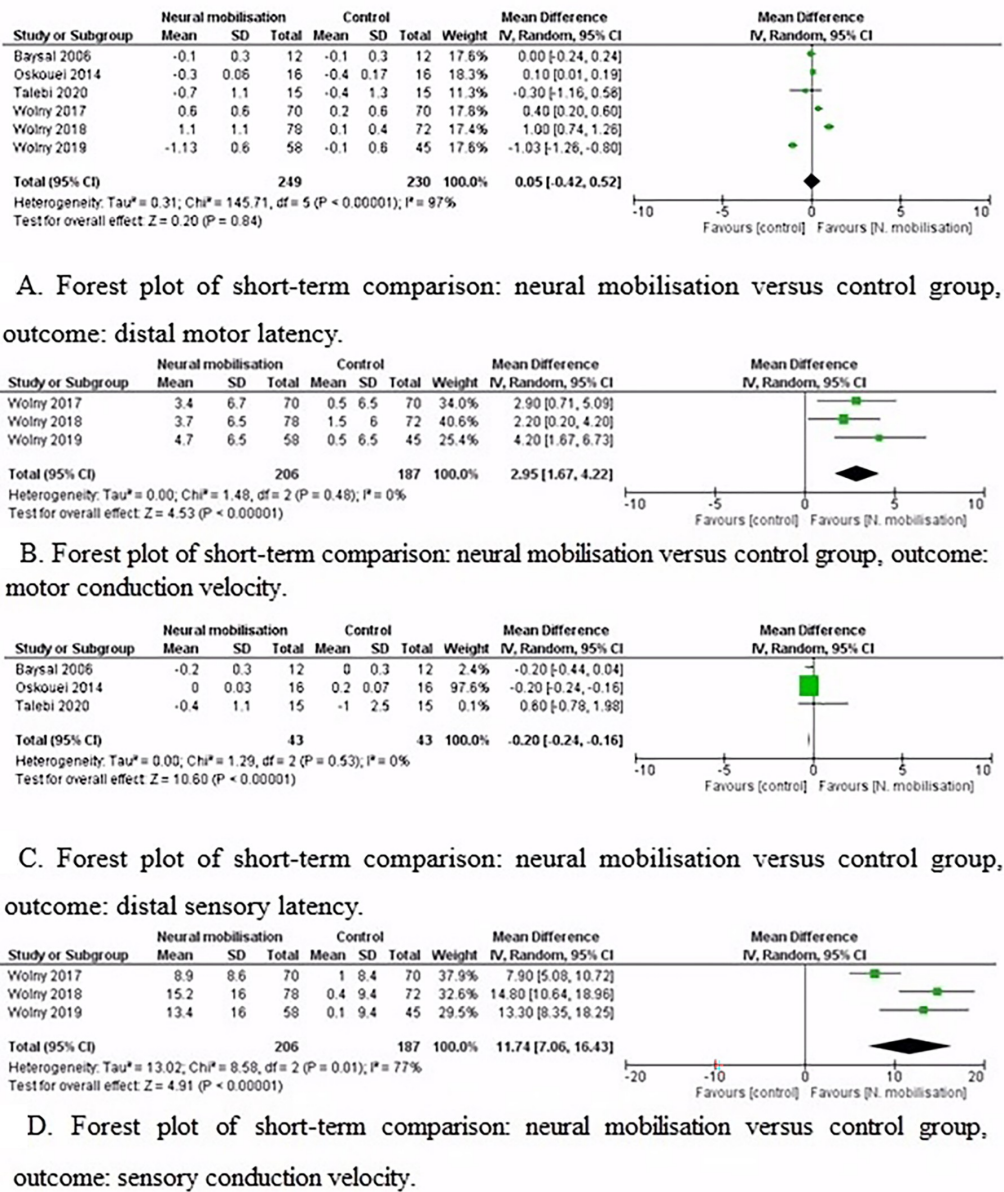
Forest plots: Neural mobilisation versus control group.

Pooled results showed that neural mobilisation did not improve distal motor latency in the short-term (Mean Difference (MD) [95% CI] = 0.05 metre per second (m/s) [-0.42, 0.52]). However, there was substantial heterogeneity (I^2^ = 97%) (Fig 2A). Two hundred and forty-nine participants were involved in the neural mobilisation group, and two hundred and thirty in the control group.

Six controlled trials tested distal motor latency in the short-term. One study showed that a significant improvement was not found in distal motor latency in groups in the short-term [7]. Another study reported a decreased distal motor latency in both groups (i.e., manual therapy with neural mobilisation or electrophysical modalities) in the short-term [49]. The authors showed an improvement of the distal motor latency only for the neural mobilisation group compared to sham [50]. Similarly, one study revealed that distal motor latency was significantly improved only in the routine physiotherapy plus neural mobilisation group [54]. Also, one study reported a lower value of distal motor latency with neural mobilisation compared to the control group in the short-term [51]. Another study showed decreased distal motor latency in both groups (i.e., neural mobilisation or mechanical interface mobilisation) with no difference in the between-group comparison [52]. Overall, these studies did not improve distal motor latency in the short-term.

#### Nerve motor conduction–Motor conduction velocity

Neural mobilisation improved motor conduction velocity in short-term (MD [95% CI] = 2.95 m/s [1.67, 4.22]) with no heterogeneity (I^2^ = 0%) (Fig 2B). Two hundred and six participants were involved in the neural mobilisation group, and one hundred eighty-seven in the control group.

Three controlled trials tested motor conduction velocity in the short-term [49–51]. One study showed no significant difference post-treatment between-group comparison (i.e., manual therapy with neural mobilisation or electrophysical modalities) in motor conduction velocity [49]. Another study demonstrated a superior effect on motor conduction velocity of the neural mobilisation compared to sham after the treatment [50]. Moreover, the authors reported no significant differences between neural mobilisation and the control groups for motor conduction velocity [51]. Overall, these studies successfully improved motor conduction velocity in the short-term.

#### Nerve sensory conduction–Distal sensory latency

Neural mobilisation did not improve distal sensory latency in the short-term (MD [95% CI] = -0.20 m/s [-0.24, 0.16]) with no heterogeneity (I^2^ = 0%) (Fig 2C). Forty-three participants were involved in each group.

Three controlled trials analysed the distal sensory latency in the short-term [7, 52, 54]. One study revealed that the treatment combinations were effective in all groups, but there was no significant difference in the between-group comparison [7]. Moreover, there are significant differences within groups for group 1 (splinting and neural mobilisation) and group 3 (splinting, neural mobilisation, and ultrasound therapy) considering the baseline versus immediately after the treatment period and baseline versus after 8 weeks follow-up [7]. Another study showed no significant improvement in distal sensory latency for the mechanical interface group. In the nerve mobilisation group, there was a significant improvement in distal sensory latency. Moreover, there was no significant difference between the two groups in distal sensory latency immediately after the treatment period (p > 0.05) [52]. Finally, one study found that there was no significant change in distal sensory latency in the control group (routine physiotherapy) or treatment group (routine physiotherapy plus neural mobilisation group) [54]. These studies did not improve distal sensory latency in the short-term treatment period.

#### Nerve sensory conduction–Sensory conduction velocity

Neural mobilisation improved sensory conduction velocity in the short-term (MD [95% CI] = 11.74 m/s [7.06, 16.43]) with considerable heterogeneity (I^2^ = 77%) (Fig 2D). Two hundred and six participants were involved in the neural mobilisation group, and one hundred and eighty-two in the control group.

Three controlled trials tested sensory conduction velocity in the short-term [49–51]. One study showed that in the manual therapy group (i.e., neural mobilisation), sensory conduction velocity was increased by 34%. Still, there was no change in nerve sensory conduction in the electrophysical modalities group [49]. The authors detected a superior effect on sensory conduction velocity of the neural mobilisation compared to sham after the treatment [50]. Also, another study identified a greater effect favoured neural mobilisation in sensory conduction velocity after ten weeks of treatment (neural mobilisation group: 38.3 m/s, SD = 11.1 vs. control group: 25.9 m/s, SD = 7.72, p < .01) [51]. Overall, these studies were successful in improving sensory conduction velocity in the short-term.

### Descriptive analysis

#### Studies ineligible for pooling

Median nerve cross-sectional area was measured in two studies, but one study did not have control group data [53]. Three outcomes (median nerve signal intensity, sciatic nerve stiffness, and median nerve integrity) were measured from individual studies with no chance of performing a meta-analysis [23, 43, 44, 46]. One study measuring sensory conduction velocity was ineligible for pooling because of the lack of control group data [42].

#### Risk of bias and overall quality of evidence

**Fig 3:**
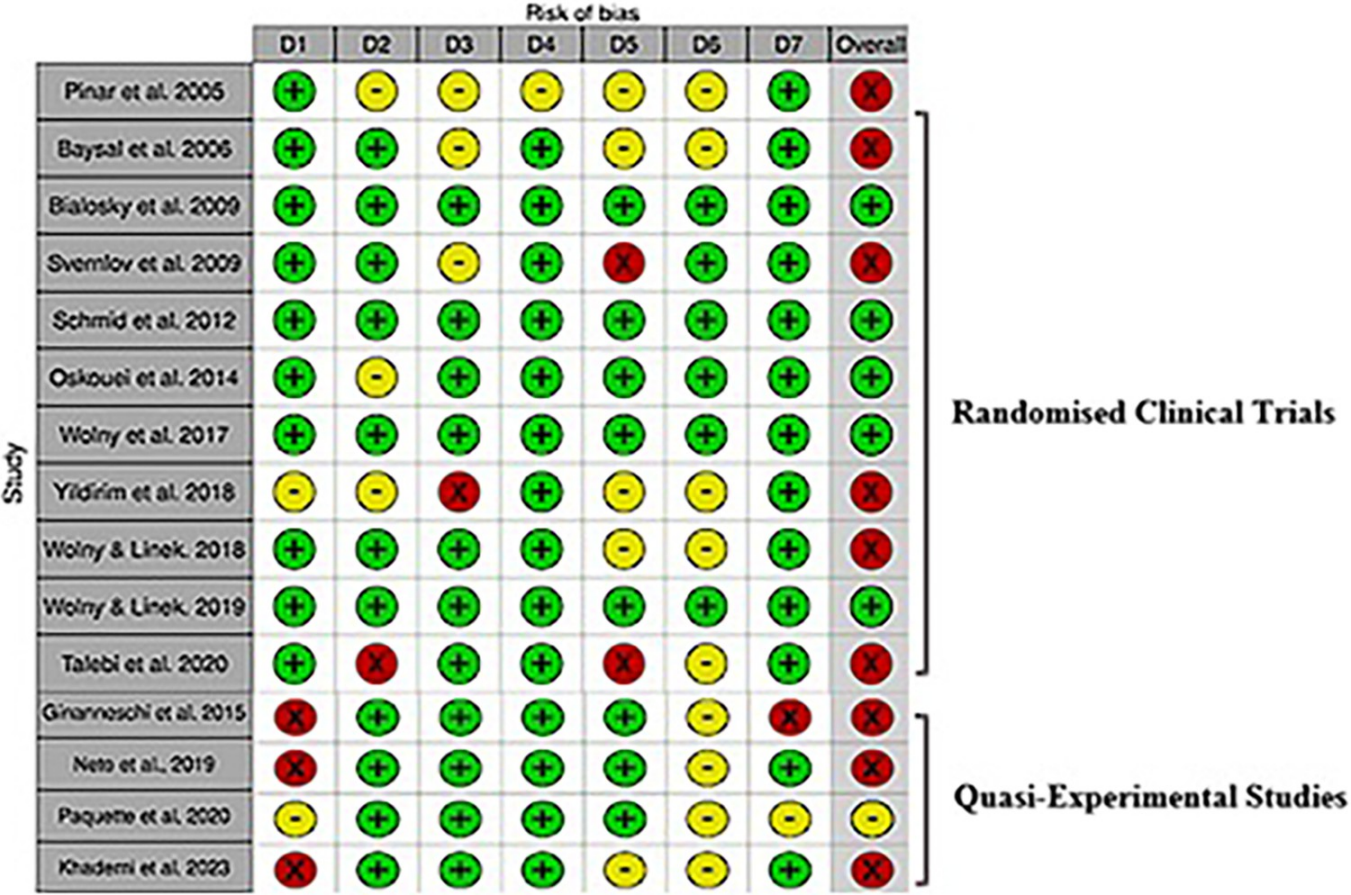
Risk of bias for included controlled trials and quasi-experimental studies.

According to the overall evaluation of the risk of bias of the controlled trials included, the risk of bias tool indicated that six articles had a high risk of bias [7, 45, 46, 48, 50, 52] and five had a low risk of bias [23, 47, 49, 51, 54] (Fig 3). Most studies scored low risk of bias in domains of random sequence generation, allocation concealment, blinding of participants and personnel, blinding of outcome assessment and other sources of bias. A high risk of bias was found frequently in incomplete outcome data. Further information in relation to the risk of bias in controlled trials and motivation for judgments can be found in S4 and S5 Files. Our results revealed that the three studies presented a low risk of bias in the domains of bias due to the selection of participants, bias in the classification of interventions, bias due to deviations from intended interventions, and bias due to missing data. Moreover, three of the four quasi-experimental studies had a serious risk of bias due to the confounding domain, and in the domain of bias in the measurement of outcomes, all studies present a moderate risk of bias. The overall classification showed that of the four quasi-experimental studies, one had a moderate risk of bias [44], and three had a serious risk of bias [42, 43, 53] (Fig 3).

We considered the quality of evidence very low for three pooled outcomes (cross-sectional area, distal motor latency, distal sensory latency, and sensory conduction velocity) and moderate for motor conduction velocity (Table 2).

**Table 2:** GRADE summary of findings.

Neural mobilisation for peripheral neuropathic pain compared to control				
Population: adults (> 18 years old) with peripheral neuropathic pain Intervention: neural mobilisation Comparison: sham, active or inactive control				
Outcomes	Mean difference (95% CI) between neural mobilisation and control	Number of participants (studies)	Confidence in effect estimate	Rating
**Cross-sectional area**	Not estimated	41 NM + KT = 10 NM = 21 (2)	⨁◯◯◯ Very low	• 1 for risk of bias, • 1 for imprecision • 1 publication bias
**Distal motor latency**	0.05 (-0.42 to 0.52) p = 0.84	479 NM = 249; control = 230 (6)	⨁◯◯◯ Very low	• 1 for risk of bias, • 1 for inconsistency, • 1 for imprecision
**Motor conduction velocity**	2.95 (1.67 to 4.22) p < 0.00001)	393 NM = 206; control = 187 (3)	⨁⨁⨁◯ Moderate	• 1 for imprecision
**Distal sensory latency**	-0.20 (-0.24 to 0.16) p < 0.00001	86 NM = 43; control = 43 (3)	⨁◯◯◯ Very low	• 1 for risk of bias, • 1 for imprecision • 1 publication bias
**Sensory conduction velocity**	11.74 (7.06 to 16.43) p < 0.00001	393 NM = 206; control = 187 (3)	⨁◯◯◯ Very low	• 1 for risk of bias, • 2 for inconsistency, • 1 for imprecision • 1 publication bias

Note: CI = Confidence Interval; GRADE, Grading of Recommendations, Assessment, Development, and Evaluations; KT = kinesiotaping; NM = Neural Mobilisation.

## Discussion

This systematic review investigated the effects of neural mobilisation on nerve function and nerve structure of patients with peripheral neuropathic pain. Utilising nerve conduction tests such as electroneuromyography and imaging exams like ultrasonography and magnetic resonance imaging emerged as the predominant methods for evaluating nerve structure and function in this context. Nearly all studies (86%) investigated the effects of neural mobilisation in patients with carpal tunnel syndrome. The median cross-sectional area improvement should be interpreted cautiously since only a high-risk-of-bias study assessed this outcome using neural mobilisation in both groups treated. Neural mobilisation improves motor and sensory conduction velocity in the short-term despite the lack of improvement in distal motor latency in the short-term and distal sensory latency immediately after the treatment period. The improvement in the motor conduction velocity was rated with moderate quality of evidence, and the other outcomes were rated with very low quality of evidence. Despite most studies showing promising results, only a minority (33%) were considered to have a low risk of bias, suggesting a need for cautious interpretation.

We recognise the strengths and limitations of the present review. To the best of our knowledge, this is the first study that investigated the effect of neural mobilisation on the nerve structure and function of patients with peripheral neuropathic pain. We included only controlled trials and quasi-experimental studies in the systematic review as they provided the best evidence on the effectiveness of neural mobilisation treatment in the nerve function and nerve structure in peripheral neuropathic pain patients. However, including experimental studies that encompass not only neural mobilisation but also other forms of therapy may hamper the identification of the particular effect of the intervention. Nonetheless, the trials in the conservative treatment of radicular pain commonly use a combination of therapeutic options. Including studies with patients with radicular pain without radiculopathy in this systematic review may represent a shortcoming to the nerve function and structure assessment.

Although our search strategy captured several eligible studies, limited evidence was available on the change of nerve cross-sectional area outcome, with studies predominately providing data on our secondary outcomes (nerve conduction tests). The current body of evidence highlights this research area has focused on patients with carpal tunnel syndrome, and only one study has investigated the sciatic nerve. Less than half (n = 7, 50%) of the included studies were published in the last five years, and the investigation in this field needs to implement methodological improvements since 64% of the studies had a high or serious risk of bias.

Neural mobilisation potentially reduces the cross-sectional area of the nerve. Our review found two studies supporting the decrease of the cross-sectional area as a marker of improvement after neural mobilisation in patients with carpal tunnel syndrome [46, 53]. Previous studies described a cross-sectional area reduction after surgical procedures for patients with carpal tunnel syndrome [55–57]. Thus, the positive sonography outcome after neural mobilisation is likely due to the favourable clinical findings previously demonstrated in many systematic reviews [19, 20, 22]. Furthermore, a notable relationship exists between the nerve cross-sectional area and nerve conduction studies in patients with carpal tunnel syndrome [58, 59].

Nerve conduction studies are helpful to investigate motor and sensory conduction velocity in patients with radiculopathies. The current investigation found that neural mobilisation improves motor and sensory conduction velocity in the short-term. We confirmed the positive effect of neural mobilisation on nerve conduction velocity described in two previous systematic reviews that focussed on carpal tunnel syndrome [60, 61], using a robust meta-analysis with the change from baseline and expanded the findings to the improvement of the cross-sectional area of the median nerve. Moreover, another systematic review found very low-quality evidence that neural mobilisation did not affect distal motor latency in patients with carpal tunnel syndrome [62], similar to our findings. Thus, neural mobilisation leads to a partial recovery of nerve function in patients with carpal tunnel syndrome and possibly in other peripheral neuropathies. The improvement in the nerve conduction velocity may represent a remyelination process after the therapeutic since the conduction velocity evaluates the demyelination of the large-diameter fibres. The current systematic review expands on the effect of neural mobilisation on nerve function and nerve structure for two other clinical conditions. One study described improved nerve conduction velocity of patients with cubital tunnel syndrome who had impairment in the baseline assessment submitted to elbow brace, neural mobilisation, or clinical information interventions [48].

In clinical practice, the findings from this systematic review suggest that neural mobilisation may be an intervention for patients with peripheral neuropathic pain, particularly those with carpal tunnel syndrome. Improving motor and sensory conduction velocity in the short term indicates a potential benefit in promoting nerve recovery. Clinicians should consider incorporating neural mobilisation into their treatment plans for these patients, keeping in mind the limitations of the current evidence, including the predominance of studies focused on carpal tunnel syndrome and the high risk of bias in many studies. The observed reduction in nerve cross-sectional area after neural mobilisation in carpal tunnel syndrome patients highlights a potential positive impact on nerve structure. However, given the limited research on other peripheral neuropathies and the need for high-quality, well-designed studies to minimise bias, clinicians should approach the integration of neural mobilisation into practice with a balanced consideration of the available evidence and patient-specific factors. The identified biases, such as lack of blinding and incomplete outcome data, underscore the importance of future research efforts in addressing these methodological shortcomings and enhancing the overall quality of evidence in this field.

Few studies have investigated neural mobilisation and its effectiveness in nerve structure and function of patients with peripheral neuropathic pain, considering the same aspects (patient population, technique used, outcome evaluation tool, and follow-up time). Therefore, controlled trials with detailed neural mobilisation schema measured by objective outcomes must facilitate clinicians’ decision-making. In this systematic review, the most commonly observed bias was the lack of blinding of the participant or therapist who administered the therapy, incomplete outcome data and selective reporting. Hence, future high-quality studies should be designed to minimise this bias. Finally, nerve structure and function parameters are essential in understanding how the nervous system behaviours and their changes can have various implications. These parameters revealed that certain aspects of the nerve’s physiology or signal transmission have been altered after the neural mobilisation treatment.

## Conclusion

Neural mobilisation seems to improve the cross-sectional area, albeit with very low-quality evidence, affecting the certainty of these findings. Neural mobilisation was superior to control in improving motor conduction velocity in patients with peripheral neuropathic pain with moderate quality evidence. Neural mobilisation was superior in improving sensory conduction velocity and presented similar results in distal motor and distal sensory latency compared to controls in patients with peripheral neuropathic pain based on very low-quality evidence. Caution is needed to generalise the results since most investigations focused on patients with carpal tunnel syndrome.

## Supporting information

S1 File This is the PRISMA 2020 checklist. (DOCX)

S2 File This is the search strategy. (DOCX)

S3 File This is the list of the studies. (XLSX)

S4 File This is the risk of bias of randomised clinical trials studies and motivation for judgments. (XLSX)

S5 File This is the risk of bias of randomised clinical trials studies and motivation for judgments. (DOCX)

S1 Table Reason for exclusion of full-text studies. (DOCX)

S2 Table Data extracted from each study for the meta-analysis. (DOCX)

## References

[1] Bouhassira D, Lantéri-Minet M, Attal N, Laurent B, Touboul C. Prevalence of chronic pain with neuropathic characteristics in the general population. Pain. 2008 Jun;136(3):380–7. doi: 10.1016/j.pain.2007.08.013 17888574

[2] van Hecke O, Austin SK, Khan RA, Smith BH, Torrance N. Neuropathic pain in the general population: a systematic review of epidemiological studies. Pain. 2014 Apr;155(4):654–62. doi: 10.1016/j.pain.2013.11.013 24291734

[3] Dworkin RH, Jensen MP, Gammaitoni AR, Olaleye DO, Galer BS. Symptom profiles differ in patients with neuropathic versus non-neuropathic pain. J pain. 2007 Feb;8(2):118–26. doi: 10.1016/j.jpain.2006.06.005 16949878

[4] Sterling M, Pedler A. A neuropathic pain component is common in acute whiplash and associated with a more complex clinical presentation. Man Ther. 2009 Apr;14(2):173–9. doi: 10.1016/j.math.2008.01.009 18358761

[5] Sterling M, Jull G, Vicenzino B, Kenardy J. Sensory hypersensitivity occurs soon after whiplash injury and is associated with poor recovery. Pain. 2003 Aug;104(3):509–17. doi: 10.1016/S0304-3959(03)00078-2 12927623

[6] Vicenzino B, Collins D, Wright A. The initial effects of a cervical spine manipulative physiotherapy treatment on the pain and dysfunction of lateral epicondylalgia. Pain. 1996 Nov;68(1):69–74. doi: 10.1016/S0304-3959(96)03221-6 9252000

[7] Baysal O, Altay Z, Ozcan C, Ertem K, Yologlu S, Kayhan A. Comparison of three conservative treatment protocols in carpal tunnel syndrome. Int J Clin Pract. 2006 Jul;60(7):820–8. doi: 10.1111/j.1742-1241.2006.00867.x 16704676

[8] Schmid AB, Tampin B, Baron R, Finnerup NB, Hansson P, Hietaharju A, et al. Recommendations for terminology and the identification of neuropathic pain in people with spine-related leg pain. Outcomes from the NeuPSIG working group. Pain. 2023 Aug;164(8):1693–704. doi: 10.1097/j.pain.0000000000002919 37235637 PMC10348639

[9] Moulin D, Boulanger A, Clark AJ, Clarke H, Dao T, Finley GA, et al. Pharmacological management of chronic neuropathic pain: revised consensus statement from the Canadian Pain Society. Pain Res Manag. 2014;19(6):328–35. doi: 10.1155/2014/754693 25479151 PMC4273712

[10] Finnerup NB, Attal N, Haroutounian S, McNicol E, Baron R, Dworkin RH, et al. Pharmacotherapy for neuropathic pain in adults: Systematic review, meta-analysis and updated NeuPSig recommendations. Lancet Neurol. 2015;14(2):162–73. 25575710 10.1016/S1474-4422(14)70251-0 PMC4493167

[11] National Institute for Health and Care Excellence (NICE). Low Back Pain and Sciatica in Over16s: Assessment and Management. London; 2016. 27929617

[12] Bernstein IA, Malik Q, Carville S, Ward S. Low back pain and sciatica: summary of NICE guidance. BMJ. 2017 Jan;356:i6748. doi: 10.1136/bmj.i6748 28062522

[13] Wright ME, Rizzolo D. An update on the pharmacologic management and treatment of neuropathic pain. JAAPA. 2017 Mar;30(3):13–7. doi: 10.1097/01.JAA.0000512228.23432.f7 28151738

[14] Stochkendahl MJ, Kjaer P, Hartvigsen J, Kongsted A, Aaboe J, Andersen M, et al. National Clinical Guidelines for non-surgical treatment of patients with recent onset low back pain or lumbar radiculopathy. Eur spine J Off Publ Eur Spine Soc Eur Spinal Deform Soc Eur Sect Cerv Spine Res Soc. 2018 Jan;27(1):60–75. doi: 10.1007/s00586-017-5099-2 28429142

[15] Dworkin RH, O’Connor AB, Kent J, Mackey SC, Raja SN, Stacey BR, et al. Interventional management of neuropathic pain: NeuPSIG recommendations. Pain. 2013 Nov;154(11):2249–61. doi: 10.1016/j.pain.2013.06.004 23748119 PMC4484720

[16] Khorami AK, Oliveira CB, Maher CG, Bindels PJE, Machado GC, Pinto RZ, et al. Recommendations for Diagnosis and Treatment of Lumbosacral Radicular Pain: A Systematic Review of Clinical Practice Guidelines. J Clin Med. 2021 Jun;10(11). 10.3390/jcm10112482 PMC8200038 34205193

[17] Shacklock M. Clinical neurodynamics: a new system of musculoskeletal treatment: Elsevier Health Sciences. 2005.

[18] Butler DS. The Sensitive Nervous System. Noigroup Publications, editor. 2000.

[19] Basson A, Olivier B, Ellis R, Coppieters M, Stewart A, Mudzi W. The Effectiveness of Neural Mobilization for Neuromusculoskeletal Conditions: A Systematic Review and Meta-analysis. J Orthop Sports Phys Ther. 2017 Sep;47(9):593–615. 28704626 10.2519/jospt.2017.7117

[20] Neto T, Freitas SR, Marques M, Gomes L, Andrade R, Oliveira R. Effects of lower body quadrant neural mobilization in healthy and low back pain populations: A systematic review and meta-analysis. Musculoskelet Sci Pract. 2017 Feb;27:14–22. 28637597 10.1016/j.msksp.2016.11.014

[21] Wolny T. The Use of Neurodynamic Techniques in the Conservative Treatment of Carpal Tunnel Syndrome—a Critical Appraisal of the Literature. Ortop Traumatol Rehabil. 2017 Oct;19(5):427–40. doi: 10.5604/01.3001.0010.5822 29154235

[22] Cuenca-Martínez F, La Touche R, Varangot-Reille C, Sardinoux M, Bahier J, Suso-Martí L, et al. Effects of Neural Mobilization on Pain Intensity, Disability, and Mechanosensitivity: An Umbrella Review With Meta-Meta-Analysis. Phys Ther. 2022 Jun;102(6). doi: 10.1093/ptj/pzac040 35421227

[23] Schmid AB, Elliott JM, Strudwick MW, Little M, Coppieters MW. Effect of splinting and exercise on intraneural edema of the median nerve in carpal tunnel syndrome—an MRI study to reveal therapeutic mechanisms. J Orthop Res Off Publ Orthop Res Soc. 2012 Aug;30(8):1343–50. 10.1002/jor.22064 22231571

[24] Brown CL, Gilbert KK, Brismee JM, Sizer PS, Roger James C, Smith MP. The effects of neurodynamic mobilization on fluid dispersion within the tibial nerve at the ankle: an unembalmed cadaveric study. J Man Manip Ther. 2011 Feb;19(1):26–34. doi: 10.1179/2042618610Y.0000000003 22294851 PMC3172954

[25] Gilbert KK, Roger James C, Apte G, Brown C, Sizer PS, Brismée JM, et al. Effects of simulated neural mobilization on fluid movement in cadaveric peripheral nerve sections: implications for the treatment of neuropathic pain and dysfunction. J Man Manip Ther. 2015 Sep;23(4):219–25. doi: 10.1179/2042618614Y.0000000094 26917940 PMC4727735

[26] Pesonen J, Rade M, Könönen M, Marttila J, Shacklock M, Vanninen R, et al. Normalization of Spinal Cord Displacement With the Straight Leg Raise and Resolution of Sciatica in Patients With Lumbar Intervertebral Disc Herniation: A 1.5-year Follow-up Study. Spine (Phila Pa 1976). 2019 Aug;44(15):1064–77. doi: 10.1097/BRS.0000000000003047 30985566

[27] Silvestri E, Martinoli C, Derchi LE, Bertolotto M, Chiaramondia M, Rosenberg I. Echotexture of peripheral nerves: correlation between US and histologic findings and criteria to differentiate tendons. Radiology. 1995 Oct;197(1):291–6. doi: 10.1148/radiology.197.1.7568840 7568840

[28] Kerasnoudis A, Tsivgoulis G. Nerve Ultrasound in Peripheral Neuropathies: A Review. J neuroimaging Off J Am Soc Neuroimaging. 2015;25(4):528–38. doi: 10.1111/jon.12261 25996962

[29] Beekman R, Visser LH. High‐resolution sonography of the peripheral nervous system–a review of the literature. Eur J Neurol. 2004;11(5):305–14. doi: 10.1111/j.1468-1331.2004.00773.x 15142223

[30] Stålberg E. Between genetics and biology. Is ENMG useful in peripheral neuropathy diagnosis and management? Rev Neurol (Paris). 2016 Oct;172(10):627–31. doi: 10.1016/j.neurol.2016.07.021 27638136

[31] Solders G, Andersson T, Borin Y, Brandt L, Persson A. Electroneurography index: a standardized neurophysiological method to assess peripheral nerve function in patients with polyneuropathy. Muscle Nerve. 1993 Sep;16(9):941–6. doi: 10.1002/mus.880160909 8395018

[32] Lefaucheur JP, Labat JJ, Amarenco G, Herbaut AG, Prat-Pradal D, Benaim J, et al. What is the place of electroneuromyographic studies in the diagnosis and management of pudendal neuralgia related to entrapment syndrome? Neurophysiol Clin. 2007;37(4):223–8. doi: 10.1016/j.neucli.2007.07.004 17996810

[33] Silva A, Manso A, Andrade R, Domingues V, Brandão MP, Silva AG. Quantitative in vivo longitudinal nerve excursion and strain in response to joint movement: A systematic literature review. Clin Biomech (Bristol, Avon). 2014 Sep;29(8):839–47. doi: 10.1016/j.clinbiomech.2014.07.006 25168082

[34] Dilley A, Greening J, Lynn B, Leary R, Morris V. The use of cross-correlation analysis between high-frequency ultrasound images to measure longitudinal median nerve movement. Ultrasound Med Biol. 2001 Sep;27(9):1211–8. doi: 10.1016/s0301-5629(01)00413-6 11597362

[35] Chen Y, Haacke EM, Li J. Peripheral nerve magnetic resonance imaging. F1000Research. 2019;8. 31700612 10.12688/f1000research.19695.1 PMC6820826

[36] Page MJ, McKenzie JE, Bossuyt PM, Boutron I, Hoffmann TC, Mulrow CD, et al. The PRISMA 2020 statement: an updated guideline for reporting systematic reviews. BMJ. 2021 Mar;372:n71. doi: 10.1136/bmj.n71 33782057 PMC8005924

[37] Deeks JJ, Higgins JP, Altmaan DG. Cochrane Handbook for Systematic Reviews of Interventions version 6.0. 2019. Chapter 10: Analysing data and undertaking meta-analyses | Cochrane Training.

[38] Higgins JPT, Altman DG, Gøtzsche PC, Jüni P, Moher D, Oxman AD, et al. The Cochrane Collaboration’s tool for assessing risk of bias in randomised trials. Bmj. 2011;343. doi: 10.1136/bmj.d5928 22008217 PMC3196245

[39] Jüni P, Loke Y, Pigott T, Ramsay C, Regidor D, Rothstein H. Risk of bias in non-randomized studies of interventions (ROBINS-I): detailed guidance. 2016.

[40] Minozzi S, Cinquini M, Gianola S, Gonzalez-Lorenzo M, Banzi R. The revised Cochrane risk of bias tool for randomized trials (RoB 2) showed low interrater reliability and challenges in its application. J Clin Epidemiol. 2020 Oct;126:37–44. doi: 10.1016/j.jclinepi.2020.06.015 32562833

[41] Salanti G, Del Giovane C, Chaimani A, Caldwell DM, Higgins JPT. Evaluating the quality of evidence from a network meta-analysis. PLoS One. 2014;9(7):e99682. doi: 10.1371/journal.pone.0099682 24992266 PMC4084629

[42] Ginanneschi F, Cioncoloni D, Bigliazzi J, Bonifazi M, Lorè C, Rossi A. Sensory axons excitability changes in carpal tunnel syndrome after neural mobilization. Neurol Sci Off J Ital Neurol Soc Ital Soc Clin Neurophysiol. 2015 Sep;36(9):1611–5. doi: 10.1007/s10072-015-2218-x 25896622

[43] Neto T, Freitas SR, Andrade RJ, Vaz JR, Mendes B, Firmino T, et al. Shear Wave Elastographic Investigation of the Immediate Effects of Slump Neurodynamics in People With Sciatica. J ultrasound Med Off J Am Inst Ultrasound Med. 2020 Apr;39(4):675–81. doi: 10.1002/jum.15144 31633231

[44] Paquette P, Higgins J, Gagnon DH. Peripheral and Central Adaptations After a Median Nerve Neuromobilization Program Completed by Individuals With Carpal Tunnel Syndrome: An Exploratory Mechanistic Study Using Musculoskeletal Ultrasound Imaging and Transcranial Magnetic Stimulation. J Manipulative Physiol Ther. 2020;43(6):566–78. doi: 10.1016/j.jmpt.2019.10.007 32861518

[45] Pinar L, Ada S. Can We Use Nerve Gliding Exercisesin Women With Carpal Tunnel Syndrome? Adv Ther. 2005;22(5):467–75. 16418156 10.1007/BF02849867

[46] Yıldırım P, Dilek B, Şahin E, Gülbahar S, Kızıl R. Ultrasonographic and clinical evaluation of additional contribution of kinesiotaping to tendon and nerve gliding exercises in the treatment of carpal tunnel syndrome. Turkish J Med Sci. 2018 Oct;48(5):925–32. doi: 10.3906/sag-1709-72 30384555

[47] Bialosky JE, Bishop MD, Price DD, Robinson ME, Vincent KR, George SZ. A randomized sham-controlled trial of a neurodynamic technique in the treatment of carpal tunnel syndrome. J Orthop Sports Phys Ther. 2009 Oct;39(10):709–23. doi: 10.2519/jospt.2009.3117 19801812 PMC2864088

[48] Svernlöv B, Larsson M, Rehn K, Adolfsson L. Conservative treatment of the cubital tunnel syndrome. J Hand Surg Eur Vol. 2009 Apr;34(2):201–7. doi: 10.1177/1753193408098480 19282413

[49] Wolny T, Saulicz E, Linek P, Shacklock M, Myśliwiec A. Efficacy of Manual Therapy Including Neurodynamic Techniques for the Treatment of Carpal Tunnel Syndrome: A Randomized Controlled Trial. J Manipulative Physiol Ther. 2017 May;40(4):263–72. doi: 10.1016/j.jmpt.2017.02.004 28395984

[50] Wolny T, Linek P. Neurodynamic Techniques Versus “Sham” Therapy in the Treatment of Carpal Tunnel Syndrome: A Randomized Placebo-Controlled Trial. Arch Phys Med Rehabil. 2018 May;99(5):843–54. doi: 10.1016/j.apmr.2017.12.005 29307812

[51] Wolny T, Linek P. Is manual therapy based on neurodynamic techniques effective in the treatment of carpal tunnel syndrome? A randomized controlled trial. Clin Rehabil. 2019 Mar;33(3):408–17. doi: 10.1177/0269215518805213 30306805

[52] Talebi GA, Saadat P, Javadian Y, Taghipour M. Comparison of two manual therapy techniques in patients with carpal tunnel syndrome: A randomized clinical trial. Casp J Intern Med. 2020;11(2):163–70. doi: 10.22088/cjim.11.2.163 32509244 PMC7265508

[53] Khademi S, Kordi Yoosefinejad A, Motealleh A, Rezaei I, Abbasi L, Jalli R. The sono-elastography evaluation of the immediate effects of neurodynamic mobilization technique on median nerve stiffness in patients with carpal tunnel syndrome. J Bodyw Mov Ther. 2023 Oct;36:62–8. doi: 10.1016/j.jbmt.2023.01.001 37949601

[54] Oskouei AE, Talebi GA, Shakouri SK, Ghabili K. Effects of neuromobilization maneuver on clinical and electrophysiological measures of patients with carpal tunnel syndrome. J Phys Ther Sci. 2014 Jul;26(7):1017–22. doi: 10.1589/jpts.26.1017 25140086 PMC4135187

[55] Hattori Y, Kawaguchi Y, Usami T, Waguri-Nagaya Y, Murakami H, Okamoto H. Median Nerve Recovery and Morphological Change on MRI at 24 Months after Open Carpal Tunnel Release. J hand Surg Asian-Pacific Vol. 2023 Apr;28(2):197–204. doi: 10.1142/S2424835523500212 37120302

[56] Funahashi T, Suzuki T, Hayakawa K, Nakane T, Maeda A, Kuroiwa T, et al. Visualization of the morphological changes in the median nerve after carpal tunnel release using three-dimensional magnetic resonance imaging. Eur Radiol. 2022 May;32(5):3016–23. doi: 10.1007/s00330-021-08447-y 35064311

[57] Chappell CD, Beckman JP, Baird BC, Takke A V. Ultrasound (US) Changes in the Median Nerve Cross‐Sectional Area After Microinvasive US‐Guided Carpal Tunnel Release. J Ultrasound Med. 2020;39(4):693–702. doi: 10.1002/jum.15146 31659789

[58] Ratasvuori M, Sormaala M, Kinnunen A, Lindfors N. Ultrasonography for the diagnosis of carpal tunnel syndrome: correlation of clinical symptoms, cross-sectional areas and electroneuromyography. J Hand Surg Eur Vol. 2022 Apr;47(4):369–74. doi: 10.1177/17531934211059808 34812067

[59] Aloi NF, Cluts LM, Fowler JR. Ultrasound Measurements of the Median Nerve at the Distal Wrist Crease Correlate With Electrodiagnostic Studies. Hand (N Y). 2023 Jul;18(5):765–71. doi: 10.1177/15589447211066349 34991383 PMC10336820

[60] Jiménez-Del-Barrio S, Cadellans-Arróniz A, Ceballos-Laita L, Estébanez-de-Miguel E, López-de-Celis C, Bueno-Gracia E, et al. The effectiveness of manual therapy on pain, physical function, and nerve conduction studies in carpal tunnel syndrome patients: a systematic review and meta-analysis. Int Orthop. 2022 Feb;46(2):301–12. doi: 10.1007/s00264-021-05272-2 34862562 PMC8782801

[61] Zaheer SA, Ahmed Z. Neurodynamic Techniques in the Treatment of Mild-to-Moderate Carpal Tunnel Syndrome: A Systematic Review and Meta-Analysis. J Clin Med. 2023 Jul;12(15). doi: 10.3390/jcm12154888 37568290 PMC10419623

[62] Núñez de Arenas-Arroyo S, Cavero-Redondo I, Torres-Costoso A, Reina-Gutiérrez S, Álvarez-Bueno C, Martínez-Vizcaíno V. Short-term Effects of Neurodynamic Techniques for Treating Carpal Tunnel Syndrome: A Systematic Review With Meta-analysis. J Orthop Sports Phys Ther. 2021 Dec;51(12):566–80. doi: 10.2519/jospt.2021.10533 34784245

