# Supplementary material for: Neural mobilisation effects in nerve function and nerve structure of patients with peripheral neuropathic pain: A systematic review with meta-analysis
Source: PLoS One. 2024 Nov 8;19(11):e0313025. doi: 10.1371/journal.pone.0313025 (PMC11548838; doi:10.1371/journal.pone.0313025)
Supplement: S2 File — (DOCX) [file pone.0313025.s002.docx]

**S2 File**. Search Strategy

MEDLINE via OVID

1. exp Peripheral Nervous System Diseases/

2. Conservative Treatment/

3. Rehabilitation/

4. exp Physical Therapy Modalities/

5. Pain Management/

6. exp Musculoskeletal Manipulations/

7. ((nerve or neural or neurodynamic or nervous) adj3 (mobili#ation or glid* or tension or stretch or slump or physical therapy or physiotherapy or therapy or treatment or modalit*)).mp

8. or/2-7

9. exp Diagnostic Imaging/

10. exp Diagnostic Techniques, Neurological/

11. exp Electrodiagnosis/

12. electroneuromyography.mp

13. nerve conduction tests.mp

14. or/9-13

15. 1 and 8 and 14

EMBASE via OVID

1. exp Peripheral Nervous System Diseases/

2. Conservative Treatment/

3. Rehabilitation/

4. exp Physical Therapy Modalities/

5. Pain Management/

6. exp Musculoskeletal Manipulations/

7. ((nerve or neural or neurodynamic or nervous) adj3 (mobili#ation or glid* or tension or stretch or slump or physical therapy or physiotherapy or therapy or treatment or modalit*)).mp

8. or/2-7

9. exp Diagnostic Imaging/

10. exp Diagnostic Techniques, Neurological/

11. exp Electrodiagnosis/

12. electroneuromyography.mp

13. nerve conduction tests.mp

14. or/9-13

15. 1 and 8 and 14

CINAHL via EBSCO

1. (MH Peripheral Nervous System Diseases)

2. (MH Conservative Treatment)

3. (MH Rehabilitation)

4. (MH Physical Therapy Modalities)

5. (MH Pain Management)

6. (MH Musculoskeletal Manipulations)

7. ((TI nerve OR neural OR neurodynamic OR nervous) N3 (mobili?ation OR glid* OR tension OR stretch OR slump OR "physical therapy" OR physiotherapy OR therapy OR treatment OR modalit*)) OR ((TI nerve OR neural OR neurodynamic OR nervous) N3 (mobili?ation OR glid* OR tension OR stretch OR slump OR "physical therapy" OR physiotherapy OR therapy OR treatment OR modalit*))

8. S2 OR S3 OR S4 OR S5 OR S6 OR S7

9. (MH Diagnostic Imaging)

10. (MH Diagnostic Techniques, Neurological)

11. (MH Electrodiagnosis)

12. (TI electroneuromyography) OR (AB electroneuromyography)

13. (TI nerve conduction tests) OR (AB nerve conduction tests)

14. S9 OR S10 OR S11 OR S12 OR S13

15. #1 AND #8 AND #14

COCHRANE

1. MeSH descriptor: [Peripheral Nervous System Diseases] explode all trees

2. MeSH descriptor: [Conservative Treatment] explode all trees

3. MeSH descriptor: [Rehabilitation] explode all trees

4. MeSH descriptor: [Physical Therapy Modalities] explode all trees

5. MeSH descriptor: [Pain Management] explode all trees

6. MeSH descriptor: [Musculoskeletal Manipulations] explode all trees

7. ((nerve:ti,ab,kw OR neural:ti,ab,kw OR neurodynamic:ti,ab,kw OR nervous:ti,ab,kw) NEAR/3 (mobili?ation:ti,ab,kw OR glid*:ti,ab,kw OR tension:ti,ab,kw OR stretch:ti,ab,kw OR slump:ti,ab,kw OR "physical therapy":ti,ab,kw OR physiotherapy:ti,ab,kw OR therapy:ti,ab,kw OR treatment:ti,ab,kw OR modalit*:ti,ab,kw))

8. #2 OR #3 OR #4 OR #5 OR #6 OR #7

9. MeSH descriptor: [Diagnostic Imaging] explode all trees

10. MeSH descriptor: [Diagnostic Techniques, Neurological] explode all trees

11. MeSH descriptor: [Electrodiagnosis] explode all trees

12. electroneuromyography:ti,ab,kw

13. "nerve conduction tests":ti,ab,kw

14. #9 OR #10 OR #11 OR #12 OR #13

15. #1 AND #8 AND #14
