# Supplementary material for: Neural mobilisation effects in nerve function and nerve structure of patients with peripheral neuropathic pain: A systematic review with meta-analysis
Source: PLoS One. 2024 Nov 8;19(11):e0313025. doi: 10.1371/journal.pone.0313025 (PMC11548838; doi:10.1371/journal.pone.0313025)
Supplement: S5 File — (DOCX) [file pone.0313025.s005.docx]

**S5 File.** Risk of bias of randomised clinical trials studies and motivation for judgments.

| Risk of Bias | | |
| --- | --- | --- |
| Title: Can We Use Nerve Gliding Exercises in Women with Carpal Tunnel Syndrome?  Reference: Pinar et al. 2005 | | |
| Bias | Authors' judgement | Support for judgement |
| Random sequence generation (selection bias) | Low risk | “Patients were divided into 2 groups through a randomized assignment method.” |
| Allocation concealment (selection bias) | Unclear risk | Not enough information regarding this issue. |
| Blinding of participants (performance bias) | Unclear risk | Not enough information regarding this issue. |
| Blinding of outcome assessment (detection bias) | Unclear risk | Not enough information regarding this issue. |
| Incomplete outcome data (attrition bias) | Unclear risk |  |
| Selective reporting  (reporting bias) | Unclear risk | No previous trial registration, but it was clear that the published report included all expected outcomes. |
| Other sources of biases | Low risk | Not found. |
| Classification | **High risk** | |

| Risk of Bias | | |
| --- | --- | --- |
| Title: Comparison of three conservative treatment protocols in carpal tunnel syndrome  Reference: Baysal et al. 2006 | | |
| Bias | Authors' judgement | Support for judgement |
| Random sequence generation (selection bias) | Low risk | “Computer-generated randomization list was created by a  biostatistician.” |
| Allocation concealment (selection bias) | Low risk | “It was given to the physiotherapy department in sealed numbered envelopes.”  “When the patients qualified to enter the study, appropriate numbered envelope was opened at the reception; the card inside indicated the patient’s allocation to a treatment group.” |
| Blinding of participants (performance bias) | Unclear risk | No mention of any attempts to blind the participants. |
| Blinding of outcome assessment (detection bias) | Low risk | “The staff who assessed the outcomes were different from the staff administering the treatments and were blinded to the type of treatment each patient had received.” |
| Incomplete outcome data (attrition bias) | High risk | “The eight dropouts are described as follows: two patients (group II) underwent surgery, two patients (group II) were lost to follow-up. In group III, two patients were lost to  follow-up, and another two patients (group III) refused electrophysiologic study due to improvement of symptoms.” |
| Selective reporting  (reporting bias) | Unclear risk | No previous trial registration, but it was clear that the published report included all expected outcomes. |
| Other sources of biases | Low risk | Not found |
| Classification | **High risk** | |

| Risk of Bias | | |
| --- | --- | --- |
| Title: A Randomized Sham-Controlled Trial of a Neurodynamic Technique in the Treatment of Carpal Tunnel Syndrome  Reference: Bialosky et al. 2009 | | |
| Bias | Authors' judgement | Support for judgement |
| Random sequence generation (selection bias) | Low risk | “Randomization was computer generated, with group assignment maintained in sealed, sequentially numbered, opaque envelopes.” |
| Allocation concealment (selection bias) | Low risk | “(…) group assignment maintained in sealed, sequentially numbered, opaque envelopes.” |
| Blinding of participants (performance bias) | Low risk | “(…) group assignment maintained in sealed, sequentially numbered, opaque envelopes.” |
| Blinding of outcome assessment (detection bias) | Low risk | “A licensed physical therapist (J.E.B.) performed all baseline  assessments (with the exception of the NCS, which was performed by K.R.V.) and all post randomization assessments until the final session at 3 weeks.” |
| Incomplete outcome data (attrition bias) | Low risk | “The participant not returning for the 3-week follow-up was assigned to the NDT group providing 3-week analysis for 19/20 (95%) participants assigned to receive NDT and 20/20 (100%) participants assigned to receive the sham intervention.” |
| Selective reporting  (reporting bias) | Low risk | “The clinical trial registration number is NCT00929123.” |
| Other sources of biases | Low risk | Not found. |
| Classification | **Low risk** | |

| Risk of Bias | | |
| --- | --- | --- |
| Title: Conservative treatment of the cubital tunnel syndrome  Reference: Svernlov et al. 2009 | | |
| Bias | Authors' judgement | Support for judgement |
| Random sequence generation (selection bias) | Low risk | “Informed consent was obtained in all cases and the patients were then randomised using sequentially numbered, sealed envelopes into three groups for different treatments, Group A, Group B and Group C (Fig 1).” |
| Allocation concealment (selection bias) | Low risk | “All patients were informed about the cause of symptoms and allocated to three groups: night splinting, nerve gliding and control.” |
| Blinding of participants (performance bias) | Unclear risk | No mention of any attempts to blind the participants. |
| Blinding of outcome assessment (detection bias) | Low risk | “An occupational therapist performed all clinical assessments and instructions. Another, independent, occupational therapist at each centre evaluated the patients before and 6 months after starting the study.” |
| Incomplete outcome data (attrition bias) | High risk | “Six patients, two from each group, completed the conservative treatment program during 3 months.” |
| Selective reporting  (reporting bias) | Unclear risk | No previous trial registration, but it was clear that the published report included all expected outcomes. |
| Other sources of biases | Low risk | Not found. |
| Classification | **High risk** | |

| Risk of Bias | | |
| --- | --- | --- |
| Effect of Splinting and Exercise on Intraneural Edema of the Median Nerve in Carpal Tunnel Syndrome—An MRI Study to Reveal Therapeutic Mechanisms  Reference: Schmid et al. 2012 | | |
| Bias | Authors' judgement | Support for judgement |
| Random sequence generation (selection bias) | Low risk | “Concealed random allocation was performed by an independent investigator using sealed envelopes.” |
| Allocation concealment  (selection bias) | Low risk | “20 patients with mild to moderate CTS were randomly allocated to either night splinting or a home program of nerve and tendon gliding exercises.” |
| Blinding of participants (performance bias) | Low risk | “To verify the inter-tester reliability of the measures, a second investigator  blinded to group allocation independently evaluated all MRI scans (JE).” |
| Blinding of outcome assessment (detection bias) | Low risk | “All MRI scans were coded and an investigator blinded to the group allocation took all measurements (AS).” |
| Incomplete outcome data  (attrition bias) | Low risk | “All participants received the treatment as allocated and adhered to the prescribed exercise program and splinting regime.” |
| Selective reporting  (reporting bias) | Unclear risk | No previous trial registration, but it was clear that the published report included all expected outcomes. |
| Other sources of biases | Low risk | Not found. |
| Classification | **Low risk** | |

| Risk of Bias | | |
| --- | --- | --- |
| Effects of Neuromobilization Maneuver on Clinical and Electrophysiological Measures of Patients with Carpal Tunnel Syndrome  Reference: Oskouei et al. 2014 | | |
| Bias | Authors' judgement | Support for judgement |
| Random sequence generation (selection bias) | Low risk | “Twenty patients (32 hands) were randomly assigned to the control group (n = 16 hands), which received routine physiotherapy including rest splint, TENS, and therapeutic ultrasound, or the treatment group (n = 16 hands), which received a neuromobilization maneuver in addition to the routine physiotherapy.” |
| Allocation concealment  (selection bias) | Unclear risk | No mention of any attempts to allocation concealment was found. |
| Blinding of participants (performance bias) | Low risk | “The study was double blinded: the participants were not aware of the details of the intervention in the groups”. |
| Blinding of outcome assessment (detection bias) | Low risk | “Also the staff performing the electrophysiological measurements and analyzing the outcome measures were also blinded to the group allocations”. |
| Incomplete outcome data  (attrition bias) | Low risk | “The patients had a mean (±SD) age of 46.7±11 years and duration of CTS symptoms of 19.6±15.9 months, and they all completed the 4-week intervention protocol. The outcome measure showed a normal distribution in both groups”. |
| Selective reporting  (reporting bias) | Low risk | “In a randomized clinical trial, registered in IRCT with the number of 138903094052N1”. |
| Other sources of biases | Low risk | Not found. |
| Classification | **Low risk** | |

| Risk of Bias | | |
| --- | --- | --- |
| Title: Efficacy of Manual Therapy Including Neurodynamic Techniques for the Treatment of Carpal Tunnel Syndrome: A Randomized Controlled Trial  Reference: Wolny et al. 2017 | | |
| Bias | Authors' judgement | Support for judgement |
| Random sequence generation (selection bias) | Low risk | “Patients were randomly assigned by drawing lots with the group number. Individuals who drew the number 1 were assigned to the MT group, and those who drew number 2 were assigned to the EM group.” |
| Allocation concealment (selection bias) | Low risk | “Participants were randomly allocated to the MT group or the EM group.” |
| Blinding of participants (performance bias) | Low risk | “The procedure in which the patient drew his or her group number was supervised by a secretary who was not otherwise involved in the study.” |
| Blinding of outcome assessment (detection bias) | Low risk | “The specialists who performed the NCS were not aware of the nature of the therapy administered to participants.” |
| Incomplete outcome data (attrition bias) | Low risk | The percentage of withdrawals and dropouts was within the acceptable rate. |
| Selective reporting  (reporting bias) | Low risk | “The clinical trial registration number is ACTRN12614000367640.” |
| Other sources of biases | Low risk | Not found. |
| Classification | **Low risk** | |

| Risk of Bias | | |
| --- | --- | --- |
| Title: Ultrasonographic and clinical evaluation of additional contribution of kinesiotaping to tendon and and nerve gliding exercises in the treatment of carpal tunnel syndrome  Reference: Yildirim et al. 2018 | | |
| Bias | Authors' judgement | Support for judgement |
| Random sequence generation (selection bias) | Unclear risk | Not enough information regarding this issue. |
| Allocation concealment (selection bias) | Unclear risk | Not enough information regarding this issue. |
| Blinding of participants (performance bias) | High risk |  |
| Blinding of outcome assessment (detection bias) | Low risk | “The clinical assessment of each patient was performed by a blind  investigator (PY) and USG measurements were performed  by another blind investigator (BD).” |
| Incomplete outcome data (attrition bias) | Unclear risk | Not enough information regarding this issue. |
| Selective reporting  (reporting bias) | Unclear risk | Not enough information regarding this issue. |
| Other sources of biases | Low risk | Not found. |
| Classification | **High risk** | |

| Risk of Bias | | |
| --- | --- | --- |
| Title: Neurodynamic techniques versus “sham” therapy in the treatment of carpal tunnel  syndrome; a randomized placebo-controlled trial  Reference: Wolny & Linek, 2018 | | |
| Bias | Authors' judgement | Support for judgement |
| Random sequence generation (selection bias) | Low risk | “Eligible patients were randomly allocated to 2 parallel groups: NT or ST using a random number generator.” |
| Allocation concealment (selection bias) | Low risk | “Those who were randomly assigned “1” were in the NT group, and those who were randomly assigned “2” were in the ST group.” |
| Blinding of participants (performance bias) | Low risk | “Randomisation and allocation were performed by persons who were not otherwise involved in the trial.” |
| Blinding of outcome assessment (detection bias) | Low risk | “NCS was performed in an independent laboratory, and the staff did not know anything about the experiment.”  “The rest of the parts of the examination were conducted by  several physiotherapists who did not know anything about patients allocation.” |
| Incomplete outcome data (attrition bias) | Unclear risk | No mention of any attempts to incomplete outcome data. |
| Selective reporting  (reporting bias) | Unclear risk | Authors report that the clinical trial registration number is ACTRN12617000672358, but we could not find the record. |
| Other sources of biases | Low risk | Not found. |
| Classification | **High risk** | |

| Risk of Bias | | |
| --- | --- | --- |
| Title: Is manual therapy based on neurodynamic techniques effective in the treatment of carpal tunnel syndrome? A randomized controlled trial.  Reference: Wolny & Linek, 2019 | | |
| Bias | Authors' judgement | Support for judgement |
| Random sequence generation (selection bias) | Low risk | “The allocation was made before the data collection began using a random number generator computer programme.” |
| Allocation concealment (selection bias) | Low risk | “Those who were randomly assigned ‘1’ were placed in the experimental group, and those who were randomly assigned ‘2’ were placed in the control group.” |
| Blinding of participants (performance bias) | Low risk | “Group assignments were sealed in opaque envelopes. Randomization and allocation were performed by two research assistants who were not otherwise involved in the trial.” |
| Blinding of outcome assessment (detection bias) | Low risk | “The nerve conduction study was performed in an independent laboratory as a standard procedure, and staff were not informed about the conducted  experiment.” |
| Incomplete outcome data (attrition bias) | Low risk | “The whole protocol accomplished with completed data from 103 participants (Figure 1). Thus, the final analysis involved 103 participants (58 in experimental and 45 in control group).” |
| Selective reporting  (reporting bias) | Low risk | Authors report that the clinical trial registration number is ACTRN12617000672358. |
| Other sources of biases | Low risk | Not found. |
| Classification | **Low risk** | |

| Risk of Bias | | |
| --- | --- | --- |
| Title: Comparison of two manual therapy techniques in patients with carpal tunnel syndrome: A randomized clinical trial  Reference: Talebi et al. 2020 | | |
| Bias | Authors' judgement | Support for judgement |
| Random sequence generation (selection bias) | Low risk | “Randomization was carried out by a simple random allocation (figure 1).” |
| Allocation concealment (selection bias) | High risk | “Patients were alternately assigned to a group as they were identified.” |
| Blinding of participants (performance bias) | Low risk | “The participants were blinded for both grouping and treatment methods.” |
| Blinding of outcome assessment (detection bias) | Low risk | “The examiner collecting the outcome measures before and after treatment procedures and the data analyst were unaware of the assigned treatment.” |
| Incomplete outcome data (attrition bias) | High risk | “However, 9 patients failed to complete all the outcome measures yielding 30 patients in the final analysis.” |
| Selective reporting  (reporting bias) | Unclear risk | Authors report that the clinical trial registration number is 201508182851N4, but we could not find the record. |
| Other sources of biases | Low risk | Not found. |
| Classification | **High risk** | |
