# Supplementary material for: Neural mobilisation effects in nerve function and nerve structure of patients with peripheral neuropathic pain: A systematic review with meta-analysis
Source: PLoS One. 2024 Nov 8;19(11):e0313025. doi: 10.1371/journal.pone.0313025 (PMC11548838; doi:10.1371/journal.pone.0313025)
Supplement: S1 Table — (DOCX) [file pone.0313025.s006.docx]

| **S1 Table.** Reason for exclusion of full text studies. | |
| --- | --- |
| **Trial (author, year)** | **Reasons for exclusion** |
| Bardak (1) et al. 2009 | Inappropriate outcomes |
| Heebner (2) et al. 2008 | Inappropriate outcomes |
| Horment-Lara (3) et al. 2015 | Inappropriate outcomes |
| Persson, Carlsson, Carlsson (4) 1997 | Inappropriate patient population |
| Rozmaryn et al. (5) 1998 | Inappropriate outcomes |
| Soyupek et al. (6) 2012 | Inappropriate intervention |
| Wolny et al. (7) 2016 | Inappropriate outcomes |
| Gonzalez et al. (8) 2016 | Inappropriate patient population |
| Horng et al. (9) 2011 | Inappropriate outcomes |
| Paquette et al. (10) 2015 | Inappropriate study design |

1. Bardak AN, Alp M, Erhan B, Paker N, Kaya B, Onal AE. Evaluation of the clinical efficacy of conservative treatment in the management of carpal tunnel syndrome. Adv Ther. 2009 Jan;26(1):107-16. doi: 10.1007/s12325-008-0134-7. Epub 2009 Jan 22. PMID: 19165436.
2. Heebner ML, Roddey TS. The effects of neural mobilization in addition to standard care in persons with carpal tunnel syndrome from a community hospital. J Hand Ther. 2008 Jul-Sep;21(3):229-40; quiz 241. doi: 10.1197/j.jht.2007.12.001. PMID: 18652967.
3. Horment-Lara G, Cruz-Montecinos C, Núñez-Cortés R, Letelier-Horta P, Henriquez-Fuentes L. Onset and maximum values of electromyographic amplitude during prone hip extension after neurodynamic technique in patients with lumbosciatic pain: A pilot study. J Bodyw Mov Ther. 2016 Apr;20(2):316-23. doi: 10.1016/j.jbmt.2015.08.006. Epub 2015 Aug 28. PMID: 27210849.
4. Persson LC, Carlsson CA, Carlsson JY. Long-lasting cervical radicular pain managed with surgery, physiotherapy, or a cervical collar. A prospective, randomized study. Spine (Phila Pa 1976). 1997 Apr 1;22(7):751-8. doi: 10.1097/00007632-199704010-00007. PMID: 9106315.
5. Rozmaryn LM, Dovelle S, Rothman ER, Gorman K, Olvey KM, Bartko JJ. Nerve and tendon gliding exercises and the conservative management of carpal tunnel syndrome. J Hand Ther. 1998 Jul-Sep;11(3):171-9. doi: 10.1016/s0894-1130(98)80035-5. PMID: 9730093.
6. Soyupek F, Yesildag A, Kutluhan S, Askin A, Ozden A, Uslusoy GA, Demirci S. Determining the effectiveness of various treatment modalities in carpal tunnel syndrome by ultrasonography and comparing ultrasonographic findings with other outcomes. Rheumatol Int. 2012 Oct;32(10):3229-34. doi: 10.1007/s00296-011-2173-7. Epub 2011 Oct 29. PMID: 22038192.
7. Wolny T, Saulicz E, Linek P, Myśliwiec A, Saulicz M. Effect of manual therapy and neurodynamic techniques vs ultrasound and laser on 2PD in patients with CTS: A randomized controlled trial. J Hand Ther. 2016 Jul-Sep;29(3):235-45. doi: 10.1016/j.jht.2016.03.006. Epub 2016 Apr 16. PMID: 27094495.
8. Gonzalez-Suarez C, NathleenDizon J, Cua R, et al. Determination of the longitudinal median nerve mobility in different neurodynamic techniques. Hand Therapy. 2016;21(1):16-24. doi:10.1177/1758998315617784
9. Horng YS, Hsieh SF, Tu YK, Lin MC, Horng YS, Wang JD. The comparative effectiveness of tendon and nerve gliding exercises in patients with carpal tunnel syndrome: a randomized trial. Am J Phys Med Rehabil. 2011 Jun;90(6):435-42. doi: 10.1097/PHM.0b013e318214eaaf. PMID: 21430512.
10. Paquette P, Lamontagne M, Higgins J, Gagnon DH. Repeatability and Minimal Detectable Change in Longitudinal Median Nerve Excursion Measures During Upper Limb Neurodynamic Techniques in a Mixed Population: A Pilot Study Using Musculoskeletal Ultrasound Imaging. Ultrasound Med Biol. 2015 Jul;41(7):2082-6. doi: 10.1016/j.ultrasmedbio.2015.03.015. Epub 2015 Apr 11. PMID: 25868536.
