# Supplementary material for: Neural mobilisation effects in nerve function and nerve structure of patients with peripheral neuropathic pain: A systematic review with meta-analysis
Source: PLoS One. 2024 Nov 8;19(11):e0313025. doi: 10.1371/journal.pone.0313025 (PMC11548838; doi:10.1371/journal.pone.0313025)
Supplement: S2 Table — (DOCX) [file pone.0313025.s007.docx]

**S2 Table 1.** Values of short-term comparison: neural mobilisation versus control group, outcome: distal motor latency.

| **Study** | **Neural mobilisation** | | | **Control** | | | **Weight** | **Mean Difference** |
| --- | --- | --- | --- | --- | --- | --- | --- | --- |
|  | **Mean** | **SD** | **Total** | **Mean** | **SD** | **Total** |  | **IV, Random, 95% CI** |
| Baysal 2006 | -0.1 | 0.3 | 12 | -0.1 | 0.3 | 12 | 17.6% | 0.00 [-0.24, 0.24] |
| Oskouei 2014 | -0.3 | 0.06 | 16 | -0.4 | 0.17 | 16 | 18.3% | 0.10 [0.01, 0.19] |
| Talebi 2020 | -0.7 | 1.1 | 15 | -0.4 | 1.3 | 15 | 11.3% | -0.30 [-1.16, 0.56] |
| Wolny 2017 | 0.6 | 0.6 | 70 | 0.2 | 0.6 | 70 | 17.8% | 0.40 [0.20, 0.60] |
| Wolny 2018 | 1.1 | 1.1 | 78 | 0.1 | 0.4 | 72 | 17.4% | 1.00 [0.74, 1.26] |
| Wolny 2019 | -1.13 | 0.6 | 58 | -0.1 | 0.6 | 45 | 17.6% | -1.03 [-1.26, -0.80] |
| **Total (95% CI)** |  |  | **249** |  |  | **230** | **100.0%** | **0.05 [-0.42, 0.52]** |

**S2 Table 2**. Values of short-term comparison: neural mobilisation versus control group, outcome: motor conduction velocity.

| **Study** | **Neural mobilisation** | | | **Control** | | | **Weight** | **Mean Difference** |
| --- | --- | --- | --- | --- | --- | --- | --- | --- |
|  | **Mean** | **SD** | **Total** | **Mean** | **SD** | **Total** |  | **IV, Random, 95% CI** |
| Wolny 2017 | 3.4 | 6.7 | 70 | 0.5 | 6.5 | 70 | 34.0% | 2.90 [0.71, 5.09] |
| Wolny 2018 | 3.7 | 6.5 | 78 | 1.5 | 6 | 72 | 40.6% | 2.20 [0.20, 4.20] |
| Wolny 2019 | 4.7 | 6.5 | 58 | 0.5 | 6.5 | 45 | 25.4% | 4.20 [1.67, 6.73] |
| **Total (95% CI)** |  |  | **206** |  |  | **187** | **100.0%** | **2.95 [1.67, 4.22]** |

**S2 Table 3.** Values of short-term comparison: neural mobilisation versus control group, outcome: distal sensory latency.

| **Study** | **Neural mobilisation** | | | **Control** | | | **Weight** | **Mean Difference** |
| --- | --- | --- | --- | --- | --- | --- | --- | --- |
|  | **Mean** | **SD** | **Total** | **Mean** | **SD** | **Total** |  | **IV, Random, 95% CI** |
| Baysal 2006 | -0.2 | 0.3 | 12 | 0 | 0.3 | 12 | 2.4% | -0.20 [-0.44, 0.04] |
| Oskouei 2014 | 0 | 0.03 | 16 | 0.2 | 0.07 | 16 | 97.6% | -0.20 [-0.24, -0.16] |
| Talebi 2020 | -0.4 | 1.1 | 15 | -1 | 2.5 | 15 | 0.1% | 0.60 [-0.78, 1.98] |
| **Total (95% CI)** |  |  | **43** |  |  | **43** | **100.0%** | **-0.20 [-0.24, -0.16]** |

**S2 Table 4.** Values of short-term comparison: neural mobilisation versus control group, outcome: sensory conduction velocity.

| **Study** | **Neural mobilisation** | | | **Control** | | | **Weight** | **Mean Difference** |
| --- | --- | --- | --- | --- | --- | --- | --- | --- |
|  | **Mean** | **SD** | **Total** | **Mean** | **SD** | **Total** |  | **IV, Random, 95% CI** |
| Wolny 2017 | 8.9 | 8.6 | 70 | 1 | 8.4 | 70 | 37.9% | 7.90 [5.08, 10.72] |
| Wolny 2018 | 15.2 | 16 | 78 | 0.4 | 9.4 | 72 | 32.6% | 14.80 [10.64, 18.96] |
| Wolny 2019 | 13.4 | 16 | 58 | 0.1 | 9.4 | 45 | 29.5% | 13.30 [8.35, 18.25] |
| **Total (95% CI)** |  |  | **206** |  |  | **187** | **100.0%** | **11.74 [7.06, 16.43]** |
